# Mechanical, Thermal and Rheological Properties of Polyethylene-Based Composites Filled with Micrometric Aluminum Powder

**DOI:** 10.3390/ma13051242

**Published:** 2020-03-09

**Authors:** Olga Mysiukiewicz, Paulina Kosmela, Mateusz Barczewski, Aleksander Hejna

**Affiliations:** 1 Institute of Materials Technology, Poznan University of Technology, Piotrowo 3, 61-138 Poznań, Poland; olga.mysiukiewicz@put.poznan.pl; 2 Department of Polymer Technology, Gdańsk University of Technology, Narutowicza 11/12, 80-233 Gdańsk, Poland; paulina.kosmela@pg.edu.pl

**Keywords:** linear low-density polyethylene, particle-shaped filler, aluminum, composite, mechanical properties, rheology, crystallization

## Abstract

Investigations related to polymer/metal composites are often limited to the analysis of the electrical and thermal conductivity of the materials. The presented study aims to analyze the impact of aluminum (Al) filler content (from 1 to 20 wt%) on the rarely investigated properties of composites based on the high-density polyethylene (HDPE) matrix. The crystalline structure, rheological (melt flow index and oscillatory rheometry), thermal (differential scanning calorimetry), as well as static (tensile tests, hardness, rebound resilience) and dynamic (dynamical mechanical analysis) mechanical properties of composites were investigated. The incorporation of 1 and 2 wt% of aluminum filler resulted in small enhancements of mechanical properties, while loadings of 5 and 10 wt% provided materials with a similar performance to neat HDPE. Such results were supported by the lack of disturbances in the rheological behavior of composites. The presented results indicate that a significant content of aluminum filler may be introduced into the HDPE matrix without additional pre-treatment and does not cause the deterioration of composites’ performance, which should be considered beneficial when engineering PE/metal composites.

## 1. Introduction

Initially, the term polymer composites referred to the materials composed of various short or continuous fibers bound together by an organic polymer matrix. They were designed to efficiently transfer loads between phases and provide high stiffness and strength with relatively low weight [1]. Therefore, their original purpose was to show superior mechanical performance [2]. Nowadays, the term polymer composites has evolved and, despite some inaccuracies, it is generally used to describe materials which consist of one or more fillers and one, or a blend of, polymers. Except for enhancing mechanical performance, the manufacturing of polymer composites may be aimed at providing other specific properties, e.g., thermal or electric conductivity, reduced flammability, corrosion or chemical resistance, reduced density, renewable nature, biodegradability or reduced cost [3,4,5,6,7].

The use of particle-shaped inorganic filler has gained ground in the scientific research. The use of thermally stable materials, such as mineral or metal particles, has become attractive from an application point of view. They are not as susceptible to mechanical and thermal degradation during melt processing conducted with high shear rates, resulting in the final beneficial properties of the composites. Moreover, the application of particle-shaped fillers with suitably selected particle size distribution allows the manufacture of highly filled composite materials without significantly reduced processability [8,9], which, in the case of the same content of fibrous fillers, provides severe production limitations [10].

Multiple research groups have investigated the incorporation of aluminum or its derivatives into polymer matrices [11,12]. The majority of the works were aimed at providing electrical or thermal conductivity, with few examples where other properties were investigated [13,14]. Bigg [15] noted that the aspect ratio of applied aluminum fibers showed a significant influence on composites’ resistivity. For the aspect ratio of 24, the loading of 6 vol% induced the composite’s conductivity, while, for spherical conductor particles, 38 vol% was required. Regarding mechanical performance, only a simple decrease in tensile strength with rising content of filler was noted. Similarly, Tamvan [16] focused mainly on the enhancement of the thermal conductivity of polyethylene with the addition of aluminum, with only observations related to the drop in tensile properties. On the other hand, Pinto and Jiménez-Martín [17] noted only an increase in hardness of composites, while the main focus was on the determination of the percolation threshold. Boudenne et al. [18] analyzed the impact of content (from 10% to 60%) and size (average size 8 or 44 μm) of aluminum fillers on the thermal properties of polypropylene-based composites. Thermal diffusivity and conductivity were increased with the filler loading. Stronger enhancement of these parameters was noted for the larger particle size of the filler, which was ascribed to the slightly increased crystallinity of the polypropylene phase, due to the nucleating effect of the filler. However, except electric and thermal conductivity, other properties have to be taken into account when developing new materials. Among them are the above-mentioned mechanical properties and rheological characteristics, which are very important for the processing of material and its durability during further use. In the presented paper, we aim to investigate the influence of incorporation of aluminum particles into a high-density polyethylene matrix on the chemical and crystalline structure, processing, physical, mechanical, thermal, and rheological performance of obtained composites.

## 2. Materials and Methods

### 2.1. Materials

**Figure 1:**
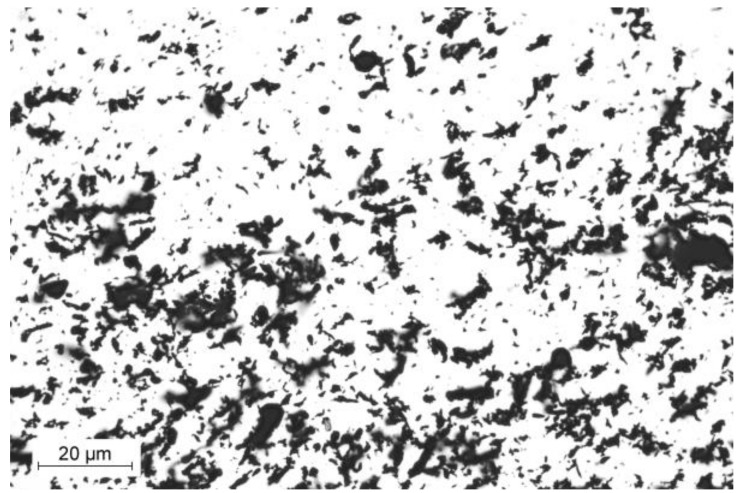
Image of aluminum (Al) powder taken using light microscopy.

High-density polyethylene (HDPE), type M300054, obtained from SABIC (Bergen op Zoom, The Netherlands), was applied as a matrix for the preparation of investigated composites. According to producer data, its density equaled 0.954 g/cm^3^, and it was characterized by a melt flow rate of 30 g/10 min (190 °C, 2.16 kg). Aluminum (Al) powder DT-90 produced by HUNTSMAN (Bergkamen, Germany) was purchased from MODELARNIA24.PL store. It was characterized by a bulk density of 2.6 g/cm^3^, and its average particle size was 65 μm. The image of the filler prepared with using light microscopy is presented in Figure 1.

### 2.2. Preparation of Polymer Composites

The composites were prepared by mixing in a molten state. To facilitate the mixing process, the HDPE pellets were pulverized into a fine powder using a Tria 25-16/TC-SL high-speed knife grinder (Tria S.p.A., Cologno Monzese, Italy). Then, the polymeric powder was preliminary mixed with 1, 2, 5, 10, or 20 wt% of aluminum powder. The mixtures were processed using a ZAMAK EH16.2D co-rotating twin-screw extruder (Zamak Mercator Sp. z o. o., Skawina, Poland) operating at 100 rpm with the maximum temperature of the process being 190 °C. The obtained materials were cooled in forced airflow and pelletized. Resulting composites were then compression molded at 170 °C and 4.9 MPa for 2 min, then kept under pressure at room temperature for another 5 min to enable solidification of the material. The unfilled HDPE was processed along with its composites. The specimens were named in reference to their filler content as PE and PE/XAl, where X stands for the filler content.

### 2.3. Measurements

The chemical structures of HDPE and its composites were determined using Fourier transform infrared spectroscopy (FTIR) analysis performed by a Nicolet Spectrometer IR200 from Thermo Fisher Scientific (Waltham, MA, USA). The device had an ATR attachment with a diamond crystal. Measurements were performed with 1 cm^−1^ resolution in the range from 4000 to 400 cm^−1^ and 64 scans.

The density of samples was measured based on the Archimedes method, as described in ISO 1183. Accordingly, all measurements were carried out at room temperature in a methanol medium.

To determine the porosity of the prepared composites, theoretical values of their density were calculated according to the simple rule of mixture, expressed by the following Equation (1):

*ρ_c_* = *ρ_m_* (1 − *ϕ*) + *ρ_f_*·*ϕ* (1)

where *ρ_C_* is the density of the composite, g/cm^3^; *ρ_m_* is the density of the matrix, g/cm^3^; *ρ_F_* is the density of the filler, g/cm^3^; φ is a volume fraction of the filler.

Using the obtained values of the density, the composites’ porosity was calculated (2):

*p* = ((*ρ_theo_* − *ρ_exp_*)/*ρ_theo_*) × 100% (2)

where *p* is the porosity of the material, %, *ρ_theo_* is the theoretical value of density, g/cm^3^ and *ρ_exp_* is an experimental value of density of composite, g/cm^3^.

The melt flow index of composites was investigated using the Zwick mFlow plastometer (Zwick Roell, Ulm, Germany) according to ISO 1133 at 190 °C, with a load of 1.2 kg.

Investigations of the rheological properties of the composites were carried using an Anton Paar MCR 301 rotational rheometer (Anton Paar, Ostfildern-Scharnhausen, Germany), with 25 mm diameter parallel plates with a 0.4 mm gap under the oscillatory mode. The experiments were conducted at 190 °C. In order to realize the dynamic oscillatory measurements, the strain sweep experiments had to be proceeded. The strain sweep experiments of all the samples were performed at 190 °C with a constant angular frequency of 10 s^−1^ in the varying strain range 0.01%–100%. The strain value, determined during the preliminary investigations and used during the frequency sweep experiments, was set as 0.5% and was located in the linear viscoelastic (LVE) region for all samples. The angular frequency used during frequency sweep measurements was in the range of 0.05–500 s^−1^. The influence of Al on the crystallization of PE in shearing conditions was realized at the same apparatus and measuring system. However, the procedure consists of preliminary melting of the material at 190 °C and following cooling down to 50 °C with a constant cooling rate of 2 °C/min, a strain of 0.1%, and angular frequency 10 s^−1^.

The tensile strength and elongation at break were estimated following ASTM D638. Tensile tests were performed on a Zwick/Roell Z020 (Zwick Roell, Ulm, Germany) apparatus with a cell load capacity of 20 kN at a constant speed of 50 mm/min.

Shore hardness type D was estimated using Zwick 3131 durometer following PN-ISO 868 (Zwick Roell, Ulm, Germany).

The rebound resilience was determined with a Schob-type pendulum Gibitre Rebound Check following the ISO 4662 standard. Each evaluation was prepared for seven test specimens.

The dynamic mechanical analysis was conducted on a DMA Q800 TA Instruments apparatus (TA Instruments, New Castle, DE, USA). Samples with dimensions of 40 × 10 × 2 mm were loaded with variable sinusoidal deformation forces in the single cantilever bending mode at the frequency of 1 Hz under the temperature rising rate of 4 °C/min, ranging the temperature from −100 to 100 °C.

To determine the crystallization and melting temperatures, as well as the crystalline structure of analyzed composites, differential scanning calorimetry (DSC), was applied. The 5 mg samples were placed in aluminum crucibles with pierced lids. They were heated from 20 to 250 °C with a heating rate of 10 °C/min and then cooled back to the initial temperature at a cooling rate of 10 °C/min. The heating/cooling cycle was performed twice in order to erase the thermal history of the polymers during the first heating. The measurements were conducted using a Netzsch 204F1 Phoenix apparatus (Netzsch, Selb, Germany), in an inert atmosphere of nitrogen. The crystallinity degree *X_cr_* of the samples was calculated using Formula (3):

*X_cr_* = Δ*H_m_*/((1 − *ϕ*) × Δ*H_m100%_*)) × 100% (3)

where Δ*H_m_* is the melting enthalpy of a sample, Δ*H_m100%_* is the melting enthalpy of 100% crystalline polyethylene, Δ*H_m100%_* = 293.6 J/g [19], *φ* is the filler weight fraction.

The crystalline structure of polyethylene and the composite containing 20 wt% of aluminum powder was assessed using polarized optical microscopy. First, 20 μm sheets were cut from the samples using a Leica microtome. They were placed between two microscopic slides and put on a Linkam TMHS 600 hot plate (Linkam Scientific Instruments, Tadworth, United Kingdom). The samples were heated to 200 °C in order to erase their thermal history and rapidly cooled (cooling rate of 30 °C/min) to 130 °C. The isothermal crystallization was conducted at this temperature for 2 h. After that, the specimens were cooled to room temperature and examined using a Nikon Ecplise E400 (Nikon, Tokyo, Japan) polarized light microscope, equipped with a 10× lens and an Opta Tech digital camera (Opta-Tech, Warsaw, Poland). The images of the samples’ crystalline structure were captured and digitally processed.

## 3. Results and Discussion

### 3.1. Spectroscopic Analysis

**Figure 2:**
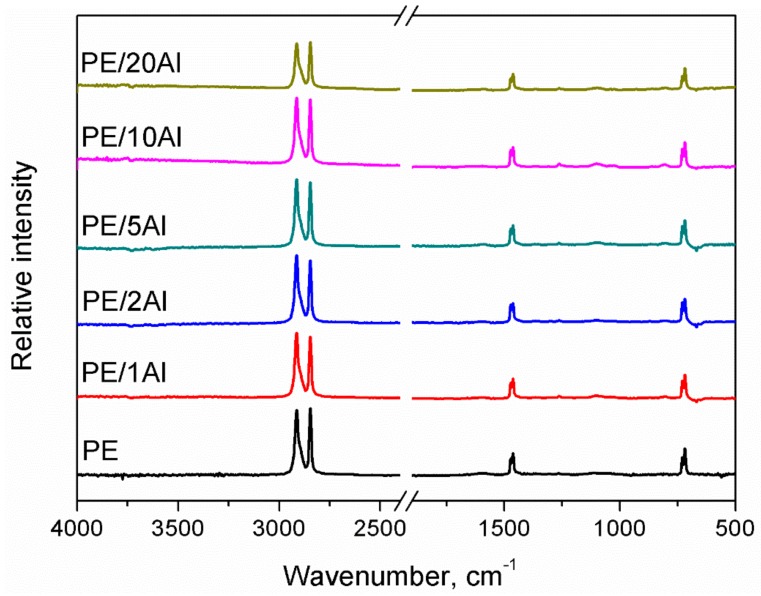
Infrared spectra of polyethylene (PE) and polyethylene/aluminum (PE/Al) composites.

In Figure 2, we present the FTIR spectra of the prepared PE/Al composites. All spectra show an appearance typical of the polyethylene applied as a matrix. Absorption bands at 2845 and 2913 cm^−1^ were associated with the symmetric and asymmetric stretching vibrations of C–H bonds. Other strong signals were noted at 1466 cm^−1^ and were attributed to the bending deformations of C–H bonds. In the range of 1100–1366 cm^−1^, there were observed weak signal characteristics for wagging and twisting deformations. Moreover, a lack of signal at 1377 cm^−1^ is typical for high-density polyethylene [20]. Peaks at 720–732 cm^−1^ were associated with the rocking vibrations of a macromolecule. As could be expected, no changes in chemical structure were noted, which points to the physical nature of potential matrix-filler interactions. The lack of an absorption band at 1740 cm^−1^, corresponding to a carbonyl group (C=O), confirms the proper realization of melt processing without degradation of polyethylene matrix [21].

### 3.2. Physical Properties

**Figure 3:**
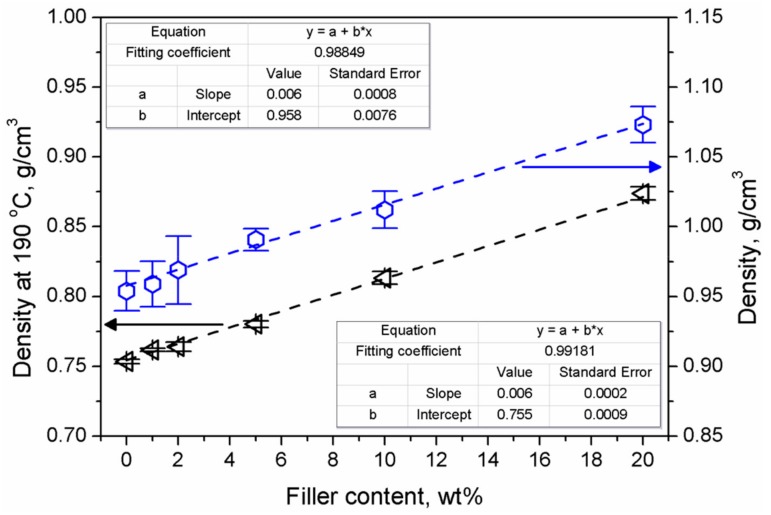
The density of the composites as a function of filler content.

Figure 3 presents experimental values of materials’ densities at ambient temperature, as well as their values at 190 °C, determined during measurements of melt flow index. As can be seen, these parameters are correlated with the content of aluminum filler characterized by notably higher density in comparison with the polymeric matrix.

**Figure 4:**
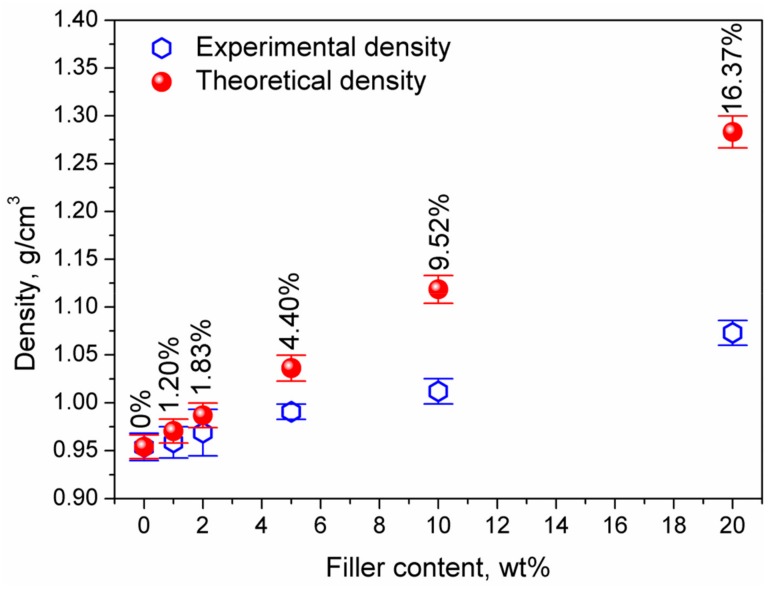
The porosity of the composites as a function of filler content.

As presented in Figure 4, the experimental and theoretical density values are different, which implicates the presence of pores in the structure. Therefore, the values of composites’ porosity, calculated according to Formula (2), are also presented in Figure 4.

Porosity is one of the most important factors influencing the mechanical performance of composite materials. The incorporation of solid particles characterized by a high free surface into the polymer matrix is very often related to the inclusion of air and the generation of the porous structure. Such a phenomenon is often observed when lignocellulosic fillers are applied, due to their moisture content and the hydrophilic character of their main components, polysaccharides [22]. The presence of moisture in the filler, which is then mixed with polymer matrix at temperatures often significantly exceeding 150 °C, results in its evaporation during processing and the generation of pores. On the other hand, even when mineral fillers are applied, obtained composites often show a partially porous structure. It can be associated with the agglomeration of filler particles and the inclusion of air inside such agglomerates, as well as entrapment of air between pellets subjected to compression molding [23,24].

### 3.3. Mechanical Properties

As mentioned above, inclusions of air are often related to the incorporation of fillers into polymer matrices, which results in the porous structure. Such a phenomenon often causes the deterioration of interfacial adhesion. Therefore, it is essential to evaluate the impact of porosity on the mechanical performance of composite materials. However, porosity in only one of the factors affecting interfacial adhesion. To comprehensively describe the strength of interfacial interactions between the matrix and filler, Kubat et al. [25] introduced the concept of the adhesion factor. It is based on the dynamic mechanical performance of composite materials. The properties of these materials depend on the performance of their constituents, matrix, filler, and interphase. Therefore, tan δ of the composite can be described with the following mathematical Formula (4):

tan *δ_c_* = *ϕ_f_* tan *δ_f_* + *ϕ_i_* tan *δ_i_* + *ϕ_m_* tan *δ_m_* (4)

where *c*, *f*, *i* and *m* subscripts are related to composite, filler, interphase, and matrix, respectively, while *φ* stands for the volume fraction of each phase.

Fillers introduced into polymer matrices, mainly mineral or metallic ones, are significantly more rigid, so their damping is very low [26]. Therefore, their influence on the adhesion factor can be neglected. Hence, Formula (4) may be rearranged into (5):

(tan *δ_c_*/tan *δ_m_*) ≅ (1 − *ϕ_f_*) × (1 + *A*) (5)

where adhesion factor (*A*) is described by the following Formula (6):

*A* = (*ϕ_i_*/(1 − *ϕ_f_*)) × (tan *δ_i_*/tan *δ_m_*) − 1 (6)

Other simplifications can be based on the fact that, compared to the share of matrix and filler, the volume fraction of interphase is meager. Also, the transcrystallinity of the interphase may be neglected, and its damping may be considered similar to the damping of the matrix. Therefore, Formula (6) may be rewritten to express the adhesion factor in terms of the relative damping of the composite and matrix, and the volume fraction of the filler. Such rearrangement leads to (7):

*A* = (1/(1 − *ϕ_f_*)) × (tan *δ_c_*/tan *δ_m_*) – 1 (7)

In Table 1, we present the values of composites’ porosity, adhesion factor, and mechanical properties affected by these parameters. Regarding the adhesion factor, its lower values are characteristic of stronger interfacial adhesion inside the composite. The increase in the A parameter results from the enhanced damping ability of the material, caused by the dissipation of energy, related to the viscoelastic nature of polymers and structural defects, such as air inclusions or “delamination” caused by insufficiently strong interfacial interactions between the matrix and filler. Negative values of adhesion factor observed on the plot are due to the neglect of the filler anisotropy, which has a slight influence on the macromolecular mobility at the filler surroundings [27].

It can be seen that for low filler loadings, a drop in the A factor is observed. Such a phenomenon indicates excellent compatibility of the polymer matrix with the introduced filler particles. When more than 2 wt% of aluminum is introduced, the increase in the A factor is observed; however, samples with 5 and 10 wt% of Al still show low beneficial values of this parameter. The significant rise can be noted for the 20 wt% loading of filler. However, due to the presence of structural defects such as porosity in the samples’ morphology, the value of the adhesion factor does not fully reflect the actual strength of the interfacial interactions.

The values of the mechanical properties of prepared composites, such as hardness, rebound resilience, tensile strength, tensile modulus and elongation at break, are collected in Table 1. All the mechanical properties of the samples change due to the addition of the metallic filler, but the course of the changes differs. Initially, when the filler content does not exceed 5 wt%, the value of tensile strength is slightly higher or equal to the one of the unfilled resin. The maximum was achieved for the 2 wt% filler loading, but the increase in tensile strength was 1 MPa only, which is rather insignificant from an application point of view. A further increase in filler content resulted in a decrease in tensile strength to 21.3 MPa (a drop of 12% compared to neat PE), measured for the sample containing 20 wt% filler.

The addition of the aluminum powder to polyethylene also changed the tensile modulus of the specimens. In the case of the composites containing up to 5 wt% of the filler, the E values decreased by approximately 50–100 MPa. The composite containing 10 wt% of the aluminum showed almost the same tensile modulus as the neat resin, while, for higher loading, the E value increased.

**Figure 5:**
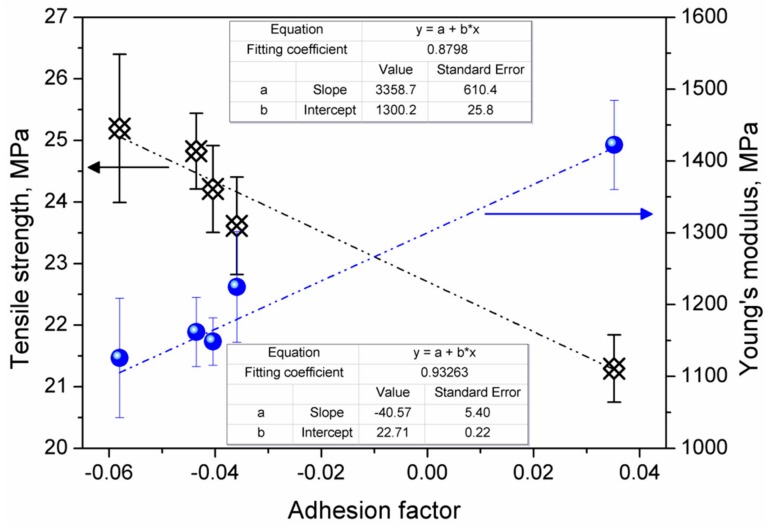
The impact of adhesion factor on the tensile strength and Young’s modulus of prepared composites.

Changes in both of these parameters were strictly associated with the structure of the composite and the strength of interfacial interactions, hence the adhesion factor, which, as mentioned above, strongly influences the mechanical performance of composite materials. In Figure 5, we present plots of tensile strength and Young’s modulus vs. adhesion factor. The highest values of tensile strength and the lowest values of modulus were noted for the lowest A parameters. Therefore, the highest tensile strength was noted for 2 wt% content of filler, when a 4% increase in strength was observed. The increase in Al content to 5 wt% caused a drop in tensile strength to the value of neat polyethylene, while, for higher contents, strength was lower than for the reference material.

On the other hand, despite the influence of the adhesion factor on tensile strength and modulus, no direct impact on the elongation at break was noted, which was more correlated to the increasing porosity of the composites. The presence of 1 wt% of aluminum powder caused a 50% decrease in elongation at break of the composite. Further increase in the filler loading resulted in the deterioration of the elongation at break value to 7.9% recorded for the 20 wt% aluminum content.

**Figure 6:**
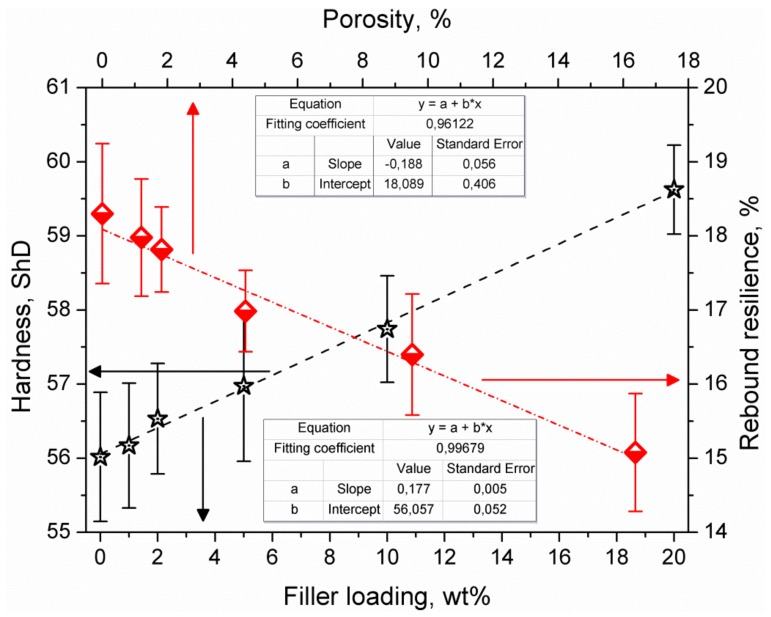
Impact of structural parameters of composites on their hardness and rebound resilience.

In Table 1, we also present values of composites’ hardness and rebound resilience. Both parameters are closely related to each other, which was already confirmed by other researchers [28]. They are also strongly related to the structure of composites, as presented in Figure 6. The dependence of rebound resilience on porosity is related to the dissipation of energy by pores and the lower ability of a porous structure to withstand and transfer stress. Such a phenomenon should also decrease the values of hardness. However, significant differences in hardness between polyethylene (~2 BHN) and aluminum (~60–100 BHN, depending on the type) overcame the impact of porosity [29,30]. Therefore, the increase in filler loading resulted in the rise in hardness.

The results of static and dynamic mechanical tests were used to calculate the brittleness of prepared materials, by following Formula (8):

*B* = 1/(*ε_b_ E’*) (8)

where *B* is the brittleness, 10^10^/%·Pa; *ε_b_* is the elongation at break, %; *E’* is the storage modulus at 25 °C, MPa.

According to the presented formula, proposed by Brostow et al. [31], brittleness can be considered as the opposition to toughness, which is the measure of stress that a material can resist before breaking. Therefore, to show the low value of brittleness, the material has to be able to withstand high stress (storage modulus included) for the wide range of strains (hence the elongation at break). For analyzed composites, brittleness was increasing with the filler loading, despite the non-linear changes of modulus. It was mainly affected by the reduction in elongation at break, which decreased from 50.0% to 7.9% when 20 wt% of Al filler was introduced to PE.

To summarize, the changes in the mechanical performance of the composites are caused by multiple factors, such as the filler dispersion in the matrix, the presence of pores, and the strength of interactions between the phases. For analyzed materials, when the filler content did not exceed 5 wt%, the particles were well dispersed in the polymeric matrix. Because of the low affinity between the polymer and the metal, they only marginally influenced tensile strength and modulus, resulting even in their enhancement. Past this point, the powder particles came into mutual contact and created rigid structures inside the polymeric matrix, which caused an increase in E value, but, on the other hand, resulted in easy crack propagation and lower tensile strength. The deterioration of elongation at break noted for all the composite samples resulted both from the presence of porosities and particle fillers, which acted as internal notches and facilitated the fracture.

### 3.4. Rheological Behavior

**Figure 7:**
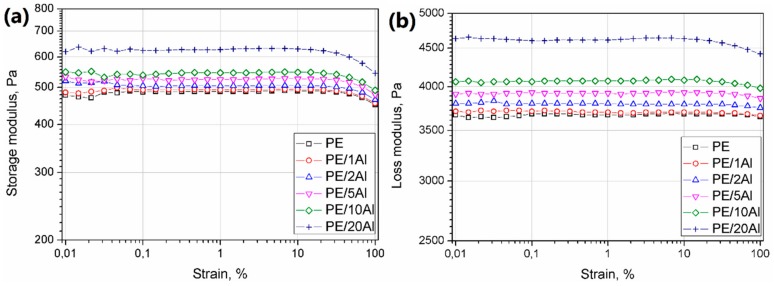
Storage (**a**) and loss (**b**) modulus vs. strain of PE and PE/Al composites.

The strain sweep experiment results presented as a variation of storage (G’) and loss (G”) modulus vs. strain (γ) are depicted in Figure 7. For both G’ and G” the same tendency was observed—an increasing amount of the filler in the composite resulted in higher modulus values. Usually, in the case of polymeric composites, the non-linearity of the plateau in low strain values at the storage modulus curve and significant shortening of the LVE region suggest the reaching of the rheological percolation threshold and the creation of a hindered 3D structure of rigid filler domains in the polymer melt [32]. The addition of up to 5 wt% of the Al powder did not influence the LVE range. Further increasing the content of the filler caused only an insignificant shortening of the LVE. This phenomenon may be interpreted as a good melt miscibility of the PE with Al.

**Figure 8:**
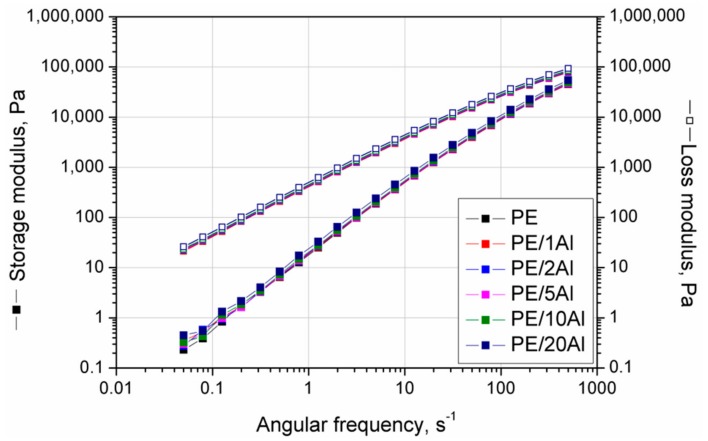
Storage and loss modulus vs. angular frequency curves of PE and PE/Al composites.

The results of frequency sweep experiments, including the influence of Al powder addition on G’ and G” vs. angular frequency (ω) curves are presented in Figure 8. For all materials tested in the same temperature conditions, dominant viscous behavior was observed (G” > G’). The incorporation of the filler showed a negligible effect on the course and slope of both G’ and G” curves. Slight gradual increases in both modules’ values, almost constant in the used during test frequency range, were noted with increasing filler content. The lack of the suppressed dependency of the ω on G’, usually observed as an inflection of the G’ curve in the low values of ω at the terminal region of the curve, suggests a lack of the agglomerated structures of the filler and its good dispersion in the polymer melt [33].

**Figure 9:**
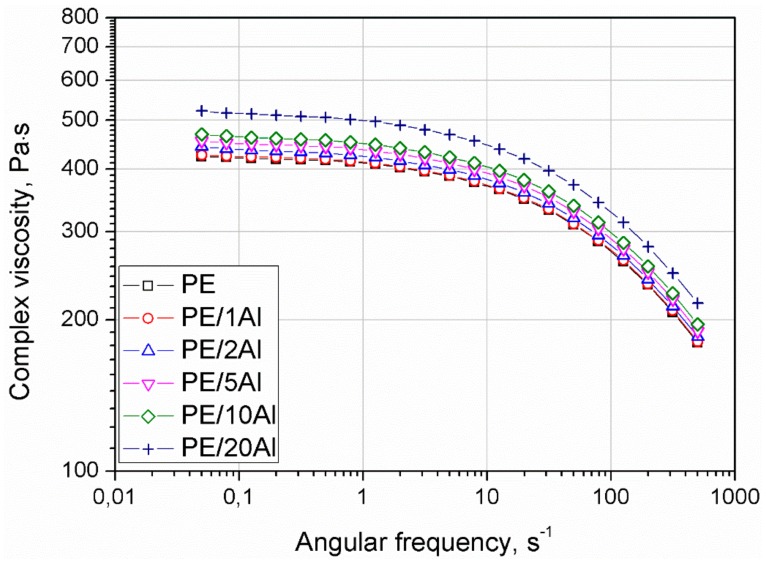
Complex viscosity as a function of angular frequency of PE and PE/Al composites.

The plots of complex viscosity (|η*|) vs. angular frequency are presented in Figure 9. All materials showed Newtonian flow behavior in the range of low ω values and shear thinning behavior. Only for samples filled with 20 wt% of the Al powder was a small deflection observed at a terminal range, corresponding to low shear rates. An increasing amount of the filler caused a gradual increase in the |η*| with a visible higher modification efficiency for the composite with the highest filler content. In contrast to the published work of Huang et al. [13], who discuss the modification of PE using nanosized Al powder, the use of Al micrometric powder did not show the significant changes of the complex viscosity curves in comparison with unmodified polyethylene. The increase in the viscosity in the considered angular frequency range is related only to the presence of well-dispersed rigid particles, rather than complex interactions between them and in the polymer–filler interface.

**Figure 10:**
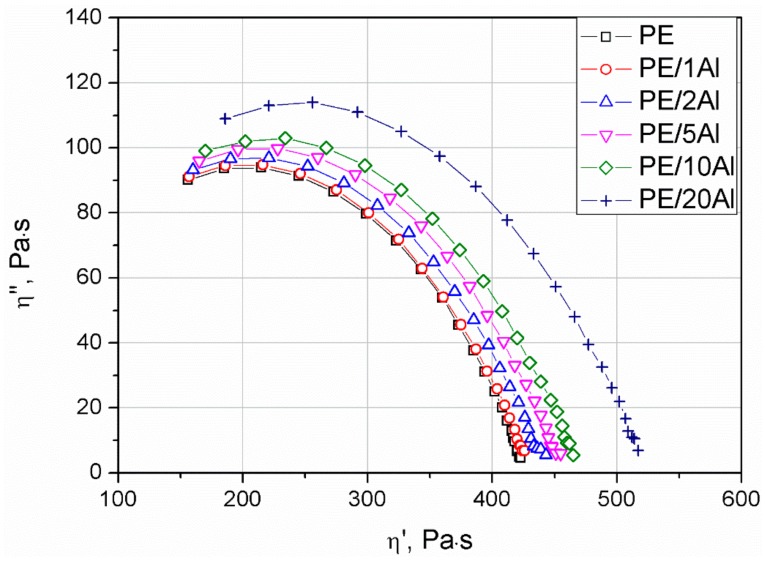
Cole–Cole plots of PE and PE/Al composites.

The miscibility of polymer blends, as well as compatibility of composite systems, may be investigated in an indirect way by analysis of Cole–Cole plots [34,35,36]. The analysis of compatibility is based on the assessment of the imaginary (η”) vs. real (η’) viscosity curve shape. If the material reveals excellent compatibility, including miscibility and proper dispersion of the filler within the polymeric matrix, the Cole–Cole plot takes the shape of a smooth semicircle. The creation of agglomerated filler structures, the presence of the immiscible domains of the additives and blended polymers or the development of cross-linked structures caused substantial deviations from the semicircle shape, usually observed as the straightening of the curve in the range of higher viscosity values. Figure 10 shows the η” presented as a function of η’. It can be stated that manufactured composites showed excellent rheological compatibility or, at least, fine dispersion of the filler in the polymeric matrix [35]. All curves showed a smooth semicircle shape without deviations in their course. The addition of even 20 wt% of the particle-shaped filler caused only increased viscosity values, without changing the character of the curve.

**Figure 11:**
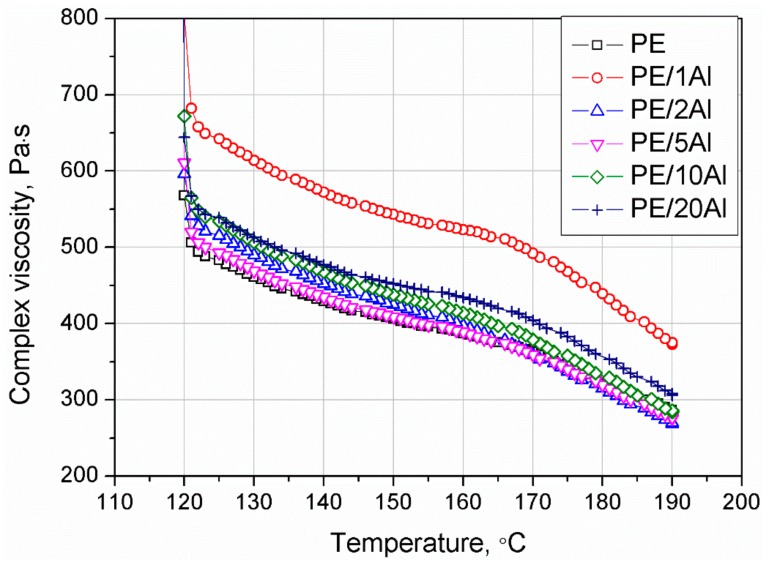
Complex viscosity of the PE and PE/Al composites as a function of temperature.

The oscillatory shear rheological evaluation in non-isothermal conditions may provide detailed supplementation of the behavior of polymers and their composites obtained by thermal analysis, about their crystallization, as well as the nucleation efficiency of their additives [37,38]. Figure 11 shows the course of the complex viscosity changes measured in steady shear conditions during the cooling of PE and its composites filled with different amounts of Al. The addition of the low amounts of Al, i.e., 5 wt% or less, only caused an increase in the melt viscosity of the composites proportional to the amount of the filler. The |η*|(T) curve was only shifted vertically about the unmodified PE curve. All considered curves showed an abrupt increase in the viscosity near 120 °C, corresponding to the crystallization of the melt, which is in good agreement with the results of DSC investigations. However, higher amounts of the filler (10 and 20 wt% of Al) also impact on the creation and further intensification of the distinct inflection of the viscosity curves at 165 °C (especially pronounced for PE/20Al sample). While the addition of 20 wt% of filler did not influence the main viscosity growth connected with the melt crystallization of PE, this phenomenon may cause several limitations during the processing of oriented products such as fibers or films. Fan and co-workers [39] studied the effect of different substrates during the crystallization of HDPE in oscillatory shearing conditions. Based on their findings that the chemical composition of metal substrate does not influence the crystallization behavior, however, the use of aluminum plates caused the earliest crystallization of the HDPE, it can be suspected that an explicit increase in the crystallinity of PE/Al composites in the range of higher temperature values may be caused by changes in the thermal conductivity of the melt.

**Figure 12:**
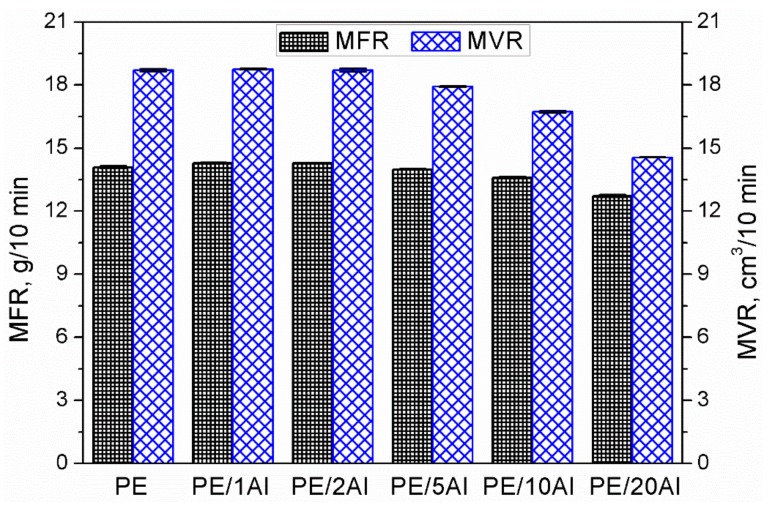
Melt mass-flow rate (MFR) and melt volume-flow rate volume (MVR) of prepared samples as a function of filler loading.

In Figure 12, there are presented values of composites’ mass (MFR), and volume (MVR) melt flow index. These parameters were used to calculate the materials’ density at 190 °C, whose values were presented above. Moreover, MFR and MVR are important technological parameters used to adjust processing parameters for a particular composition. It can be seen that the incorporation of aluminum filler did not result in a significant drop in the melt flow index, which is often observed for polymer composites [40]. Moreover, a slight increase in these parameters was noted for low contents of filler up to 2 wt%. Such an effect can be considered beneficial because it means that the application of aluminum filler does not cause difficulties during processing. The results of MFR and MVR investigations are in good agreement with discussed changes in the rheological behavior assessed by oscillatory rheological tests.

### 3.5. Thermal Properties

**Figure 13:**
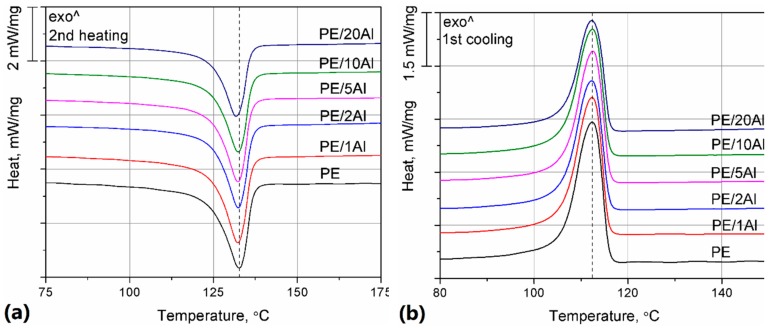
Differential scanning calorimetry (DSC) curves obtained during the second heating (**a**) and the first cooling (**b**) of the samples.

The DSC curves obtained during the second heating and the first cooling of the composites and the pure PE are presented in Figure 13. The values of melting (*T_m_*), crystallization (*T_cr_*), and onset crystallization (*T_ocr_*) temperature, as well as melting enthalpy (Δ*H_m_*) and crystallinity degree (*X_cr_*) calculated according to Formula (3), are collected in Table 2.

The thermograms presented in Figure 13 show that polyethylene, a typical semicrystalline polymer, undergoes melting of the crystalline phase in the temperature range of 110–140 °C and its melting temperature, understood as the maximum on the heating curve, is 132.7 °C. This result is in line with the literature data [11]. Polyethylene also crystallizes from the melt; the onset of crystallization temperature was established at 115.9 °C and the peak of crystallization took place at 112.4 °C. The addition of aluminum does not significantly change the melting and crystallization of the polymeric matrix. The T_cr_ and T_ocr_ values of the composite samples are almost the same as in the case of pure PE. The 0.1–0.2 °C differences can be neglected, taken into consideration the resolution of the measuring systems. It can be concluded that Al powder neither improves nor hinders the movements of polyethylene macromolecules and the creation of crystallites.

Interestingly, the melting temperature of the polymeric phase also does not considerably change due to the addition of the metallic filler. However, a small shift towards lower temperatures can be seen. This behavior can be explained by the addition of the filler, which leads to a decrease in crystallite size. The possibilities of spherulite growth are restricted due to the presence of Al particles, and the resulting finer crystalline structure presents lower T_m_ [41]. It also needs to be noted that the differences mentioned above are too low to result in any issues during the processing of the composites.

In order to further analyze the influence of aluminum on the crystallization of PE, melting enthalpy values obtained, the second heating of the samples was collected. It must be noted that ΔH_m_ values decrease gradually, from 208.4 J/g for pure PE to 166.2 J/g for the sample filled with 20 wt% of Al. The lower content of the polymer can explain the decrease in the composite samples, which is supported by the results of the crystallinity degree, calculated for the polymeric samples according to Formula (3). It can be observed that the X_cr_ values are similar, independent from the presence and amount of the filler.

Interestingly, Zhou, who studied LLDPE-based composites filled with silane-treated Al powder, observed a decrease in crystallinity. Limited possibilities of macromolecular movement explained this result due to the presence of the filler particles [41]. Therefore, it may be concluded that in the case of the studied samples, the aluminum particles do not restrain the movements of polymeric chains, presumably due to a lack of compatibility between the polymer and the filler, which was not treated with any coupling agent. On the other hand, polyethylene is a polymer presenting a relatively high crystallization rate coefficient [42]. Therefore, it is reasonable that aluminum powder, characterized by large particles (up to 100 μm) and low affinity to the polymer, will not significantly change its crystallization behavior.

### 3.6. Crystalline Structure

**Figure 14:**
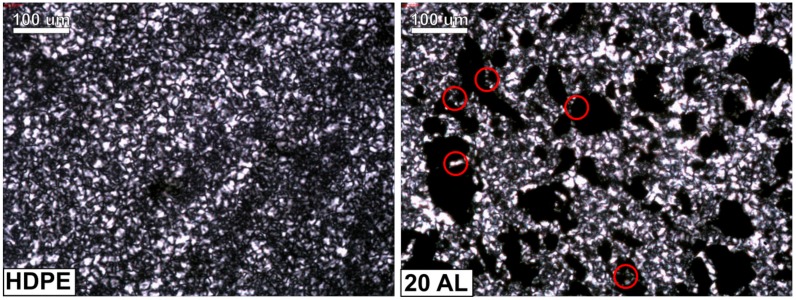
The crystalline structure of the PE (**left**) and PE/20Al composite (**right**), the red circles mark the areas where the crystallites are less developed because of spatial hindrances cause by presence of Al particles.

The images of the samples’ crystalline structure, obtained using a polarized optical microscope, are shown in Figure 14. Both samples were subjected to isothermal crystallization at 130 °C for 2 h to reveal a well-developed crystalline structure. The crystallites grown in the pure PE sample are irregular in shape, but uniform in size. The observed morphology is typical for isothermally crystallized PE [43]. The spherulites visible in the microstructure of the composite sample are very similar to the ones observed in the case of pure polyethylene. The only difference can be seen in the confined areas created by filler particles, where smaller, less-developed crystallites can be noticed (marked with red circles in Figure 14)—the growth of the crystallites is spatially limited by the Al particles in their close proximity. This observation is in line with the results of DSC, where a decrease in T_m_ in the case of the composite samples was attributed to the presence of smaller spherulites. What is more, the shape of the polyethylene spherulites does not change at the polymer–filler interface, and the transcrystalline layer cannot be seen; that is, the direction (orientation) of the growth of the crystallites is not changes by the presence of the Al particles. This observation proves that Al particles do not act as crystallization seeds for PE.

## 4. Conclusions

The presented research paper was aimed at investigating the impact of aluminum filler on the static and dynamic mechanical performance, as well as structure, thermal, and rheological properties of PE/Al composites. Research works associated with polymer/metal composites are usually focused on thermal and, most of all, electrical conductivity, with very little information related to the processing, mechanical or thermal performance of the obtained materials, which are essential from a technological point of view. Rheological tests, as well as values of mass and volume melt flow index, did not indicate significant disturbances in flowability and processing of composite polymer melts. Such an effect is very beneficial for the potential application of analyzed materials. Moreover, the incorporation of aluminum powder did not provide additional nucleating effects, so the crystallinity of the polyethylene phase was kept at a similar level, with a slight decrease by 3.4% for 2 wt% filler loading. As a result, for low contents, up to 2 wt%, small enhancements of mechanical properties were noted, while loadings up to 10 wt% provided materials with similar performance to neat PE. The presented results showed that a significant amount of aluminum filler might be introduced into the polyethylene matrix without causing the deterioration of the composites’ performance. It is also important that no additional pre-treatment of filler or enhancement of interfacial adhesion was introduced. Generally, the presented results should provide interesting and valuable insights into the engineering and preparation of polymer/metal composites.

## Figures and Tables

**Table 1:** Physico-mechanical characteristics of prepared samples.

Property	PE	PE/1Al	PE/2Al	PE/5Al	PE/10Al	PE/20Al
Porosity, %	0.00	1.20	1.83	4.40	9.52	16.37
Adhesion factor	—	−0.0436	−0.0580	−0.0404	−0.0359	0.0352
Tensile strength, MPa	24.2 ± 0.9	24.8 ± 0.6	25.2 ± 1.2	24.2 ± 0.7	23.6 ± 0.8	21.3 ± 0.6
Elongation at break, %	50.0 ± 14.9	25.6 ± 7.3	21.6 ± 4.8	17.5 ± 1.0	10.6 ± 3.5	7.9 ± 1.9
Young’s modulus, MPa	1220 ± 29	1162 ± 48	1126 ± 83	1149 ± 33	1225 ± 76	1422 ± 62
Hardness, ShD	56.0 ± 0.9	56.2 ± 0.8	56.5 ± 0.7	57.0 ± 1.0	57.7 ± 0.7	59.6 ± 0.6
Rebound resilience, %	18.3 ± 0.9	18.0 ± 0.8	17.8 ± 0.6	17.0 ± 0.5	16.4 ± 0.8	15.1 ± 0.8
*E’* at 25 °C, MPa	1507	1433	1296	1388	1766	1853
*E”* at 25 °C, MPa	90	81	72	78	97	105
tan δ at 25 °C	0.0596	0.0568	0.0557	0.0564	0.0550	0.0567
Brittleness, 10^10^/%·Pa	0.133	0.273	0.358	0.413	0.534	0.682

**Table 2:** Thermal properties of pure polyethylene and PE/Al composites determined during differential scanning calorimetry (DSC) analysis.

Sample	*T_m_*, °C	*T_cr_*, °C	*T_ocr_*, °C	Δ*H_m_*, J/g	*X_cr_*, %
PE	132.7	112.4	115.9	208.4	71.0
PE/1Al	132.3	112.4	115.8	207.1	71.3
PE/2Al	132.4	112.3	116.0	194.6	67.6
PE/5Al	132.3	112.5	116.1	196.0	70.3
PE/10Al	132.4	112.4	115.9	188.7	71.4
PE/20Al	131.7	112.3	116.1	166.2	70.8
